# Thymopentin ameliorates dextran sulfate sodium-induced colitis by triggering the production of IL-22 in both innate and adaptive lymphocytes

**DOI:** 10.7150/thno.35015

**Published:** 2019-10-12

**Authors:** Qiuhua Cao, Xinghua Gao, Yanting Lin, Chongxiu Yue, Yue Wang, Fei Quan, Zixuan Zhang, Xiaoxuan Liu, Yuan Lu, Yanling Zhan, Hongbao Yang, Xianjing Li, Di Qin, Lutz Birnbaumer, Kun Hao, Yong Yang

**Affiliations:** 1 State Key Laboratory of Natural Medicines, China Pharmaceutical University, Nanjing, Jiangsu 211198, PR China; 2 Center for New Drug Safety Evaluation and Research, China Pharmaceutical University, Nanjing, Jiangsu 211198, PR China; 3 School of Sports and Health, Nanjing sport institute, Nanjing, Jiangsu 210001, PR China; 4 Neurobiology Laboratory, National Institute of Environmental Health Sciences, Research Triangle Park, North Carolina 27709, USA, and Institute of Biomedical Research (BIOMED), Catholic University of Argentina, Buenos Aires C1107AFF, Argentina; 5 Key Lab of Drug Metabolism & Pharmacokinetics, China Pharmaceutical University, Nanjing, Jiangsu 210009, PR China

**Keywords:** thymopentin, DSS-induced colitis, lymphocytes, IL-22, gut microbiota

## Abstract

**Background:** Ulcerative colitis (UC) is a chronic inflammatory gastrointestinal disease, notoriously challenging to treat. Previous studies have found a positive correlation between thymic atrophy and colitis severity. It was, therefore, worthwhile to investigate the effect of thymopentin (TP5), a synthetic pentapeptide corresponding to the active domain of the thymopoietin, on colitis.

**Methods:** Dextran sulfate sodium (DSS)-induced colitis mice were treated with TP5 by subcutaneous injection. Body weight, colon length, colon weight, immune organ index, disease activity index (DAI) score, and the peripheral blood profile were examined. The immune cells of the spleen and colon were analyzed by flow cytometry. Histology was performed on isolated colon tissues for cytokine analysis. Bacterial DNA was extracted from mouse colonic feces to assess the intestinal microbiota. Intestinal lamina propria mononuclear cells (LPMCs), HCT116, CT26, and splenocytes were cultured and treated with TP5.

**Results:** TP5 treatment increased the body weight and colon length, decreased the DAI score, and restored colon architecture of colitic mice. TP5 also decreased the infiltration of immune cells and expression levels of pro-inflammatory cytokines such as IL-6. Importantly, the damaged thymus and compromised lymphocytes in peripheral blood were significantly restored by TP5. Also, the production of IL-22, both in innate and adaptive lymphoid cells, was triggered by TP5. Given the critical role of IL-22 in mucosal host defense, we tested the effect of TP5 on mucus barrier and gut microbiota and found that the number of goblet cells and the level of Mucin-2 expression were restored, and the composition of the gut microbiome was normalized after TP5 treatment. The critical role of IL-22 in the protective effect of TP5 on colitis was further confirmed by administering the anti-IL-22 antibody (αIL-22), which completely abolished the effect of TP5. Furthermore, TP5 significantly increased the expression level of retinoic acid receptor-related orphan receptor γ (RORγt), a transcription factor for IL-22. Consistent with this, RORγt inhibitor abrogated the upregulation of IL-22 induced by TP5.

**Conclusion:** TP5 exerts a protective effect on DSS-induced colitis by triggering the production of IL-22 in both innate and adaptive lymphocytes. This study delineates TP5 as an immunomodulator that may be a potential drug for the treatment of UC.

## Introduction

Ulcerative colitis (UC) is a chronic inflammatory gastrointestinal disorder most commonly afflicting adults aged 30-40 years impacting the quality of life 1, 2. The incidence and prevalence of UC have increased worldwide with most cases reported in northern Europe, the United Kingdom, and North America 2. However, UC has also been increasing in the East 3. UC places a heavy burden on health-care systems; the annual direct and indirect costs related to UC are estimated to be as high as $8.1-14.9 billion in the USA 4. As UC remains incurable so far, in the clinic, UC treatment has focused mainly on anti-inflammatory or immune-suppressing drugs, including 5-aminosalicylic acid, glucocorticosteroids, and immunosuppressants 5. Despite their widespread use, there is little evidence to support the use of drugs such as glucocorticosteroids in UC. Moreover, although these treatments may reduce disease symptoms temporarily, they do not prevent future bouts of the disease and have a wide array of side effects 6. New therapeutic strategies to treat UC are, therefore, urgently needed. Since UC is thought to be driven by a seemingly aberrant immune system attacking the gastrointestinal tract 7, immune-suppressive drugs are the mainstay of UC treatment. Although the topical colon presents with a hyperactive immune response, the pathology of UC is far more complicated than an abnormal immune response.

Thymic atrophy is found in many kinds of colitis models, including a Gai2^-/-^ colitis model 8, 9 and a dextran sulfate sodium (DSS)-induced colitis model 10. Our recent results also showed a marked thymic involution in severe colitis 11. Thymus, the primary lymphoid organ for T lymphocytes, is a common target organ that easily undergoes atrophy 12, 13. Thymic atrophy caused by several endogenous and exogenous factors results in an impaired release of thymus-derived T cells and in impaired host immunity development and maturation. Considering the positive relationship between thymic atrophy and colitis severity 9, it is alarming that the widely used drugs for UC are immune-suppressive drugs, which are known to induce thymic atrophy and not much effort has been directed towards restoring or replacing thymic function in these diseases.

Thymopentin (TP5), a synthetic pentapeptide corresponding to position 32-36 of thymopoietin, exhibits similar biological activity as thymopoietin responsible for phenotypic differentiation of T cells and the regulation of immune systems 14. TP5 has been clinically used for the treatment of patients with immunodeficiency diseases, such as rheumatoid arthritis, cancers, hepatitis B virus infection, and acquired immunodeficiency syndrome (AIDS) 15, 16. Despite its extensive use in the clinic, the pharmacological effects and mechanisms action of TP5 have scarcely been studied in the past 10 years. Also, the possible effect of TP5 on colitis has not been investigated. Thus, the biological activity of TP5 on thymus prompted us to investigate its effect on colitis. Herein, we discovered that TP5 ameliorates DSS-induced colitis, accompanied by markedly increased lymphocyte fraction in peripheral blood. TP5 increased the expression of IL-22 and normalized the composition of gut flora which contributed to its therapeutic effect on colitis. Our findings highlight a new therapeutic strategy to restore UC.

## Results

### TP5 alleviated DSS-induced colitis

**Figure 1:**
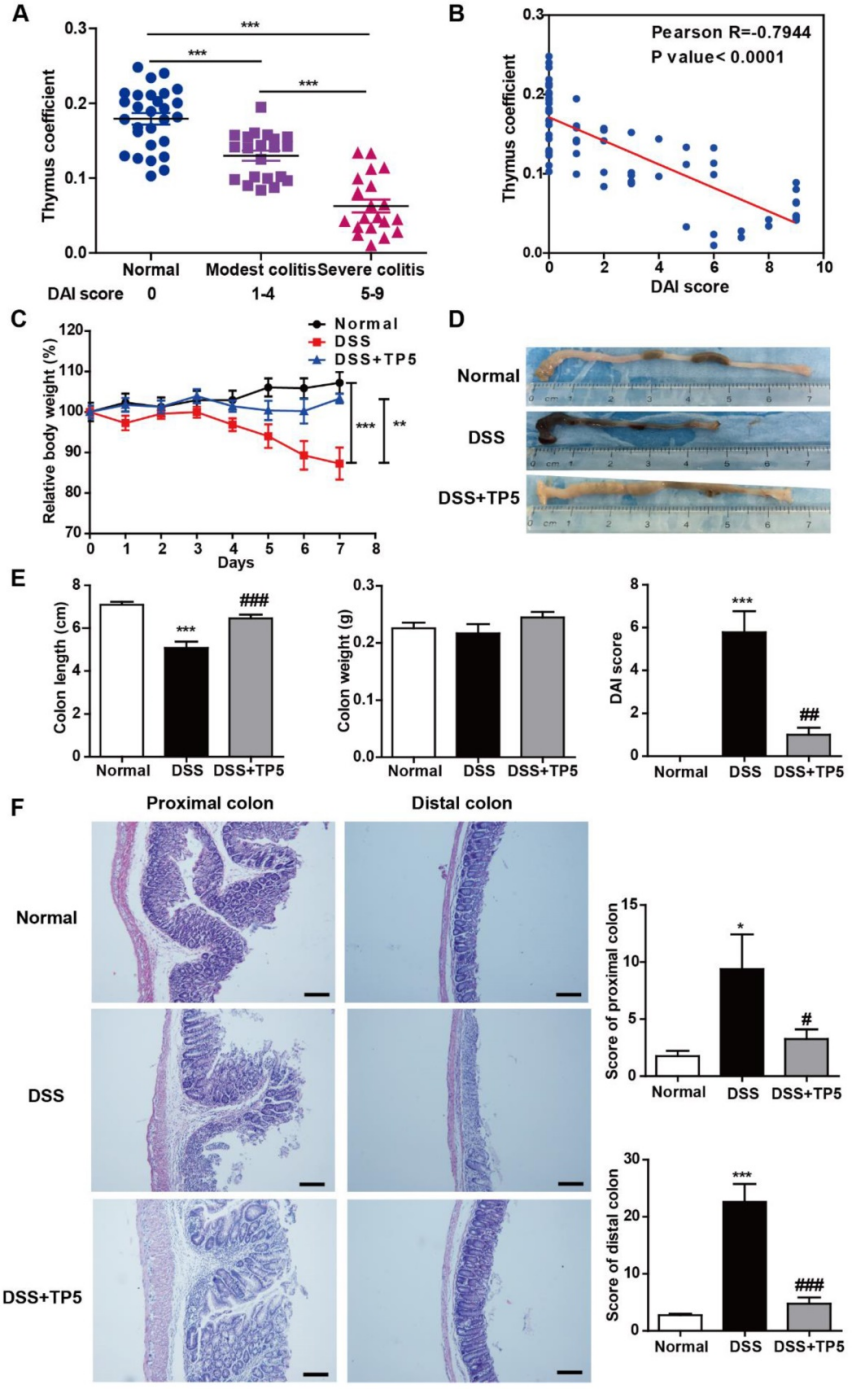
TP5 alleviated DSS-induced colitis. (A) Thymus coefficient (thymus weight/body weight × 100) in colitis with different degrees of severity. (n=20-28). (B) Correlation between thymus coefficient and DAI score. (n=69). (C) Relative body weight of mice in each group after DSS treatment. (Relative body weight = body weight/average body weight in day 0 × 100) (n=10). (D) Representative colon pictures of each group. (E) Colon length, colon weight, and DAI scores in each group. (n=9-10). (F) Representative H&E staining, pathological scores of the proximal colon and distal colon in each group. The scale bar represents 100 μm. (n=4-5). * *P* < 0.05, ** *P* < 0.01, *** *P* < 0.001, compared with the normal group; # *P* < 0.05, ## *P* < 0.01, ### *P* < 0.001, compared with the DSS group. DAI: disease activity index; DSS: dextran sulfate sodium; TP5: thymopentin. Three independent experiments were performed.

The thymus can be considered as a barometer of health, readily undergoing atrophy in a variety of infectious diseases 12. We observed thymic involution in DSS-induced colitis which was exacerbated as the severity of colitis progressed (Figure 1A), and thymus coefficient was negatively correlated to the disease activity index (DAI) score (Figure 1B). These results support the notion that promoting thymus restitution might be considered as a therapeutic approach for colitis. Remarkably, TP5, a synthetic alternative thymopoietin, dramatically blocked DSS-induced colitis, prevented the body weight loss, and increased the length of the colon and decreased the DAI score (Figure 1C-E). DSS-induced colitis was characterized by severe pathology throughout the proximal and distal colon, with extensive epithelial damage and inflammatory infiltrate. TP5 administration significantly decreased DSS-induced epithelial damage and inflammatory infiltrate and protected the integrity of the colon structure (Figure 1F). To monitor the effects of TP5 on the physiological functions in normal mice, we treated C57BL/6N mice with saline or TP5 for 7 days. TP5 had no noticeable effect on mice including body weight, colon length, and histological structure of the colon (Figure S1A-C). The weight remained unchanged in mice treated with TP5 for 14 days (Figure S1D). Collectively, these results reveal that TP5 ameliorates the development of DSS-induced colitis.

### TP5 had no direct effect on colon epithelial cells

**Figure 2:**
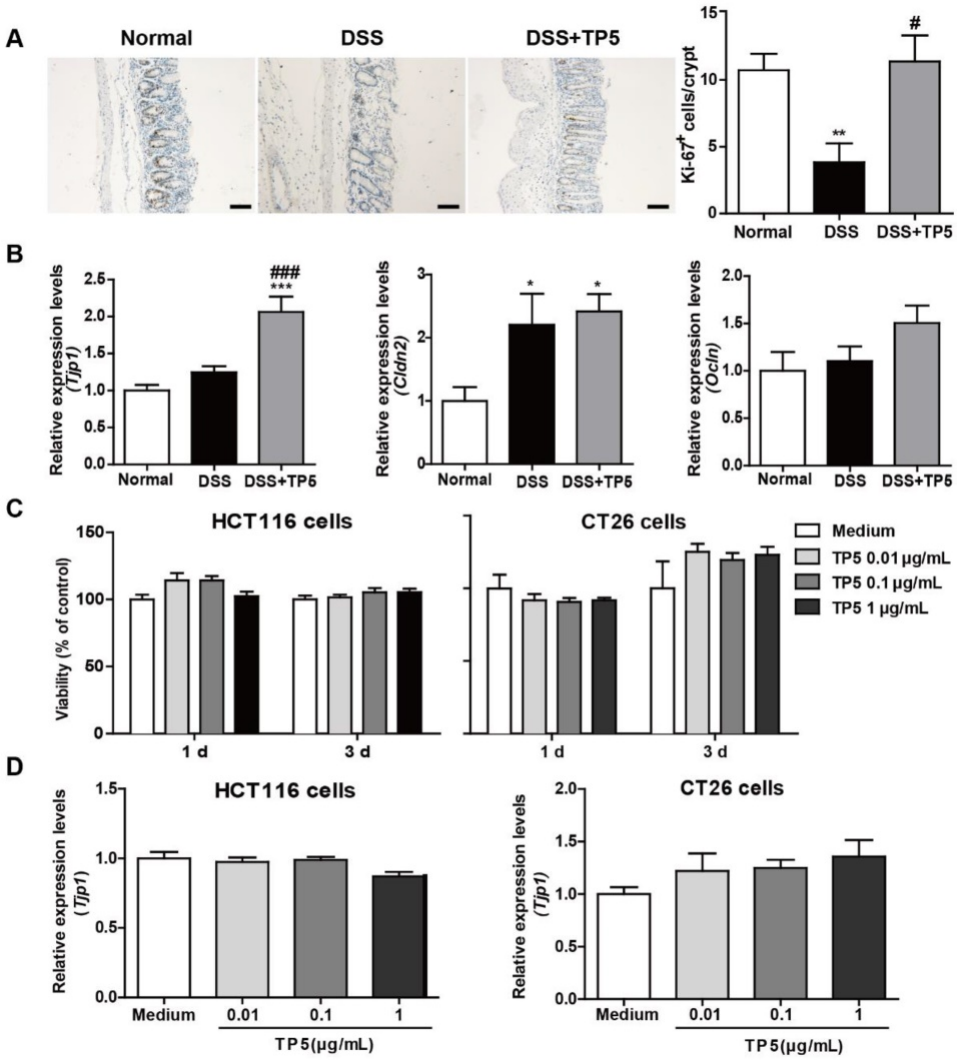
TP5 had no direct effect on colon epithelial cells. (A) Representative Ki-67 staining photographs, and mean positive Ki-67 cells counted in 20 crypts. The scale bar represents 50 μm. (n=4-5). (B) RT-PCR results of *Tjp1, Cldn2, Ocln* in mouse colons. (n=4-5). (C) Proliferation of TP5-treated HCT116 and CT26 cells. (n=6). (D) RT-PCR results for *Tjp1* in HCT116 and CT26 cells. (n=6). * *P* < 0.05, ** *P* < 0.01, *** *P* < 0.001, compared with the normal group; # *P* < 0.05, ### *P* < 0.001, compared with the DSS group. Cldn2: claudin-2; DSS: dextran sulfate sodium; Ocln: occludin; Tjp1: tight junction protein 1; TP5: thymopentin. At least two independent experiments were performed.

The epithelial layer in the gastrointestinal tract represents the first line of defense against potential enteric pathogens. Epithelial regeneration is especially important for epithelial repair 17. Consistent with the protective effect of TP5 on colitis, a significant increase in Ki-67 positive cells was observed in the colon of TP5-treated mice (Figure 2A). Intercellular junctions include the tight junctions, which contribute to the epithelial barrier 18. TP5 significantly increased the mRNA level of the *tight junction protein 1 (Tjp1)*, but not mRNA levels of other tight junction proteins like *Claudin-2 (Cldn2)* and* Occludin (Ocln)* (Figure 2B). To examine whether TP5 could directly promote the proliferation of epithelial cells, we treated two colon epithelial cell lines with TP5. Unlike the Ki-67 staining results *in vivo*, TP5 did not affect the proliferation of HCT116 or CT26 cells (Figure 2C). Also, no differences were found in *Tjp1* expression after TP5 treatment in either HCT116 or CT26 cells (Figure 2D). These results indicate that TP5 has no direct effect on colon epithelial cells.

### TP5 restored the number of peripheral lymphocytes

**Figure 3:**
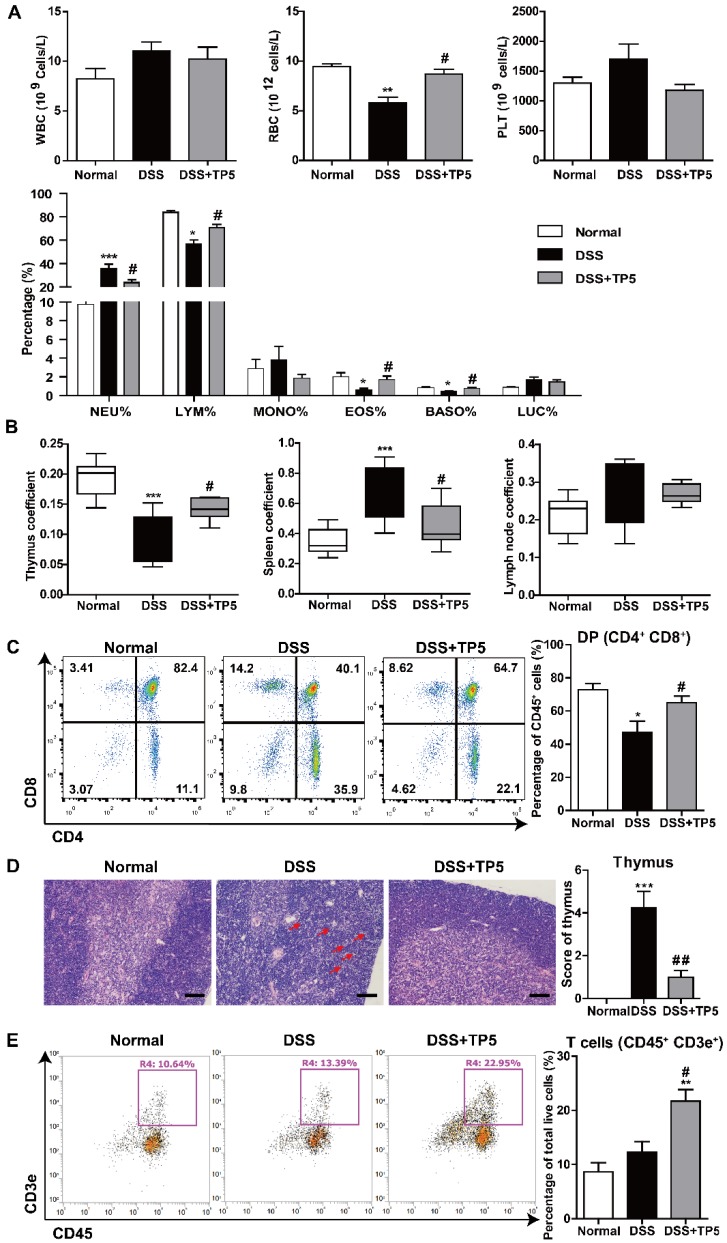
TP5 restored circulating lymphocytes. (A) Blood examination of mice, including WBC, RBC, PLT, and percentage of NEU, LYM, MONO, EOS, BASO, and LUC. (n=4-5). (B) Thymus coefficient, spleen coefficient and lymph node coefficient in mice. (n=9-10). Organ (Thymus/spleen/lymph node) coefficient = Organ (Thymus/ spleen/lymph node) weight/body weight × 100. (C) Flow cytometry analysis of double positive (DP) cells (CD45^+^, CD4^+^, CD8^+^) in the thymus. (n=4-5). (D) Representative H&E staining photographs of the thymus in each group. The arrows showed the necrotic cells. The scale bar represents 50 μm. (n=4-5). (E) Flow cytometry analysis of T cells (CD45^+^, CD3e^+^) in the spleen. (n=4-5). * *P* < 0.05, ** *P* < 0.01, *** *P* < 0.001, compared with the normal group; # *P* < 0.05, ## *P* < 0.01 compared with the DSS group. BASO: basophil; DSS: dextran sulfate sodium; EOS: eosinophils; LUC: large unstained cell; LYM: lymphocyte; MONO: monocyte; NEU: neutrophil; PLT: platelet; RBC: red blood cell; TP5: thymopentin; WBC: white blood cell. At least two independent experiments were performed.

Given the negative correlation between thymus coefficient and severity of colitis and the critical role of TP5 in the immune system, we set out to detect the effect of TP5 on circulating peripheral immune cells. We first analyzed the number and influx of circulating blood cells from the peripheral blood using an automated hematology analyzer (Siemens ADVIA2120i, Germany). DSS or TP5 treatment caused no significant changes in the number of circulating platelets (PLT), but TP5 significantly abrogated the downregulated number of red blood cells (RBC) induced by DSS, which is consistent with the protective effect of TP5 on colitic bleeding (Figure 3A). Although no significant differences were found for the total white blood cells (WBC) in all groups, the subgroups of WBC were changed. DSS caused a significant increase in neutrophils (NEU)%, while TP5 significantly decreased the proportion of NEU. Notably, the percentage of lymphocytes (LYM) was diminished in the DSS group, while TP5 significantly restored the percentage of LYM. TP5 also restored the percentages of eosinophils (EOS) and basophils (BASO). For monocytes (MONO)% and large unstained cell (LUC)%, no differences were found in all groups (Figure 3A). When mice were treated with saline or TP5 for 7 days, there were no differences in LYM%, NEU%, and MONO% (Figure S1E). However, the percentage of LYM was substantially increased in mice treated with TP5 for 14 days (Figure S1F).

Thymus involution was observed in the DSS group, which was consistent with our previous study as well as those reported by others 11, 19. As expected, TP5 significantly increased the thymus coefficient and saved the thymus from involution (Figure 3B). To examine whether TP5 alone could promote the enlargement of the thymus, we determined the thymus coefficient in TP5-treated mice. We observed that TP5 increased the thymus coefficient after 14 days of treatment but not in the mice treated for only 7 days (Figure S1G-H). It is possible that short-term use of TP5 has no apparent effect on the thymus, while long-term use can promote thymus regeneration in normal mice.

We investigated the effect of TP5 on T lymphocytes in the thymus using flow cytometry (Figure S2A). The results showed a significant decrease in the percentage of immature double positive (DP) thymocytes in the DSS group and a significant increase after TP5 administration (Figure 3C). The absolute number of total thymocytes were in line with the percentage of DP thymocytes (Figure S2B). Simultaneously, a substantial decrease of the CD4 single positive (SP) thymocytes and a slight decrease of the CD8 SP thymocytes were observed in the TP5-treated group compared with the DSS group (Figure S3B). As previously reported, thymic atrophy was found in DSS-induced colitis 10, and was accompanied with the cortical and medullary disorder in thymus 20. In our study, the hematoxylin & eosin (H&E) staining of thymus indicated that DSS induced large intercellular space, cortical and medulla structure disorder and cell necrosis, while TP5 could alleviate the detrimental effects of DSS (Figure 3D). Also, the splenomegaly induced by DSS was reduced by TP5, though no significant differences were found in the lymph node coefficient (Figure 3B). Furthermore, TP5 had no effect on spleen coefficient of normal mice (Figure S1I-J). Similar to the results in blood, TP5 increased the percentage of LYM T and B cells in the spleen (Figure 3E and S2E). There were no significant changes in NEU%, NK cell%, or the composition of CD4 and CD8 LYM in the spleen (Figure S2D). All these results indicate that TP5 restores the number of peripheral lymphocytes.

### TP5 diminished inflammation in the colon and increased IL-22 expression

**Figure 4:**
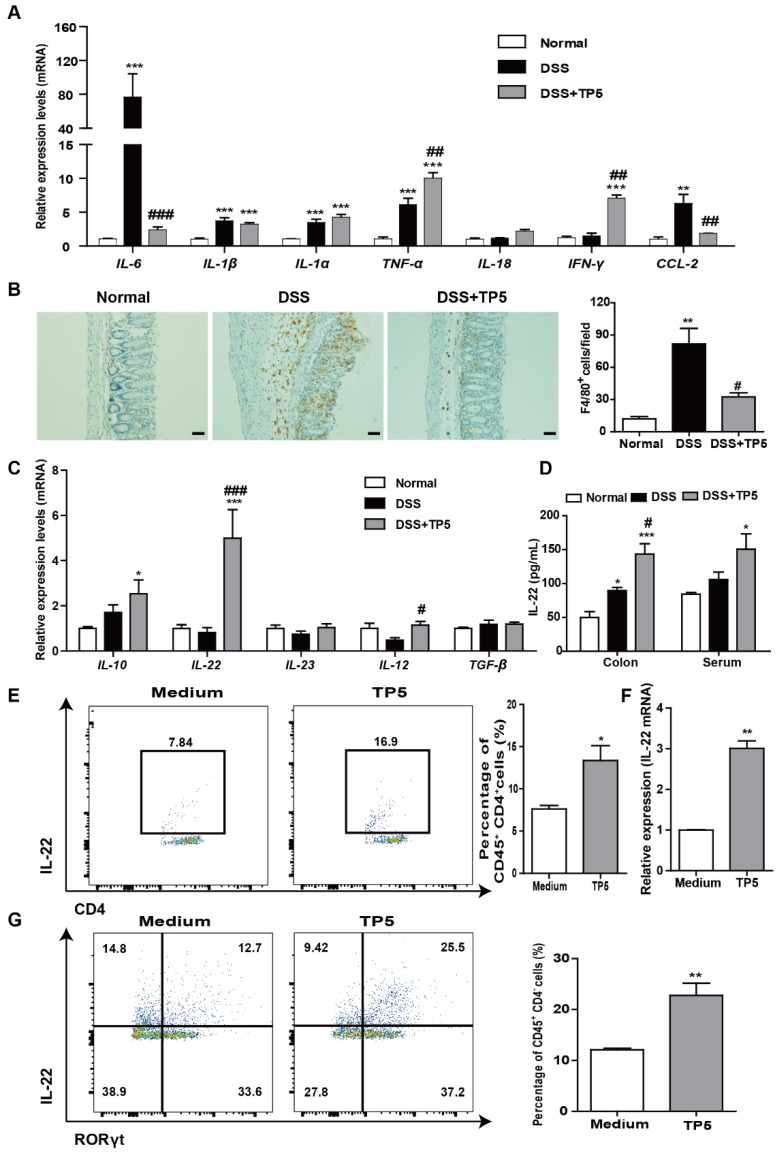
TP5 diminished inflammation in the colon and increased IL-22 expression. (A) RT-PCR results of *IL-6*, *IL-1β*, *IL-1α*, *TNF-α*, *IL-18*, *IFN-γ*, and *CCL-2* in colons of mice. (n=4-5). (B) Representative F4/80 staining photographs, and mean F4/80 positive cells counted in 5 fields. The scale bar represents 50 μm. (n=4-5). (C) RT-PCR results of *IL-10*, *IL-22*, *IL-23*, *IL-12*, and* TGF-β* in mouse colons. (n=4-5). (D) ELISA result of IL-22 in mouse colons and blood. (n=4-5). (E) Flow cytometry analysis of IL-22-producing splenocytes (CD45^+^, CD4^+^, IL-22^+^) treated with TP5 *in vitro*. (n=4). (F) RT-PCR results of *IL-22* expression induced in splenocytes by TP5 *in vitro*. (n=4). (G) Flow cytometry analysis of IL-22-producing ILC3s (CD45^+^, CD4^-^, RORγt^+^, IL-22^+^) LPMCs treated with TP5 *in vitro*. (n=4). * *P* < 0.05, ** *P* < 0.01, *** *P* < 0.001, compared with the normal group/medium group; # *P* < 0.05, ## *P* < 0.01, ### *P* < 0.001, compared with the DSS group. DSS: dextran sulfate sodium; TP5: thymopentin. At least two independent experiments were performed.

A hyperactive immune response in the colon is the hallmark of colitis. We, therefore, evaluated the effect of TP5 on inflammation-related cytokines and markers. Among the pro-inflammatory markers, DSS induced a significant increase of *IL-6* mRNA and protein levels in the colon as previously described 21, while TP5 reversed the increased expression of IL-6 almost back to normal levels (Figure 4A and S3A). For *IL-1β*,* IL-1α*, *TNF-α,* and *IL-18*, TP5 had no apparent effect. However, TP5 significantly increased the mRNA and protein levels of IFN-γ, a cytotoxic cytokine promoting not only immunomodulation but also antimicrobial activity (Figure 4A and S3A). Infiltration of immune cells in the colon is a hallmark of UC. We observed an upsurge of chemokine* CCL2* and an influx of macrophages into the colon, both of which were significantly decreased by TP5 (Figure 4A, 4B and S3B). Infiltration of T cells and NEU were also increased in the DSS group and compromised by TP5 (Figure S3C-D).

Among some other cytokines, we identified a noticeable increase of *IL-22* mRNA, and also a significant increase of *IL-10* and *IL-12* mRNAs in TP5-treated samples (Figure 4C). No differences were found in *IL-23* and *TGF-β* mRNA expression. Protein levels of IL-22 were also substantially increased by TP5 not only in the colon but also in the blood (Figure 4D). IL-22, a potent cytokine involved in tissue recovery and maintenance of barrier function, is mainly produced by T cells and group 3 innate lymphoid cells (ILC3s) 22, 23. We first isolated the splenocytes and treated them with TP5 *in vitro*. Results showed a significant increase in the percentage of IL-22^+^ CD4^+^ T cells and increased expression of *IL-22* mRNA (Figure 4E-F). Also, ILC3s of murine colon lamina propria mononuclear cells capable of producing IL-22 17 were isolated and treated with TP5 *in vitro*. Results showed increased production of IL-22^+^ ILC3s (CD45^+^ CD4^-^ RORγt^+^ IL-22^+^) in the TP5 group (Figure 4G). Besides, upregulation of IL-22 was observed in the thymus (Figure S3E) together with an increase of thymus coefficient in the group treated with TP5 for 14 days (Figure S1H). No differences in the IL-22 concentration were found in the serum and colon (Figure S3E). The data from these observations emphasize that TP5 increases the expression of IL-22, which may protect mice from colitis.

### TP5 maintained mucus barrier and normalized gut microbiota

**Figure 5:**
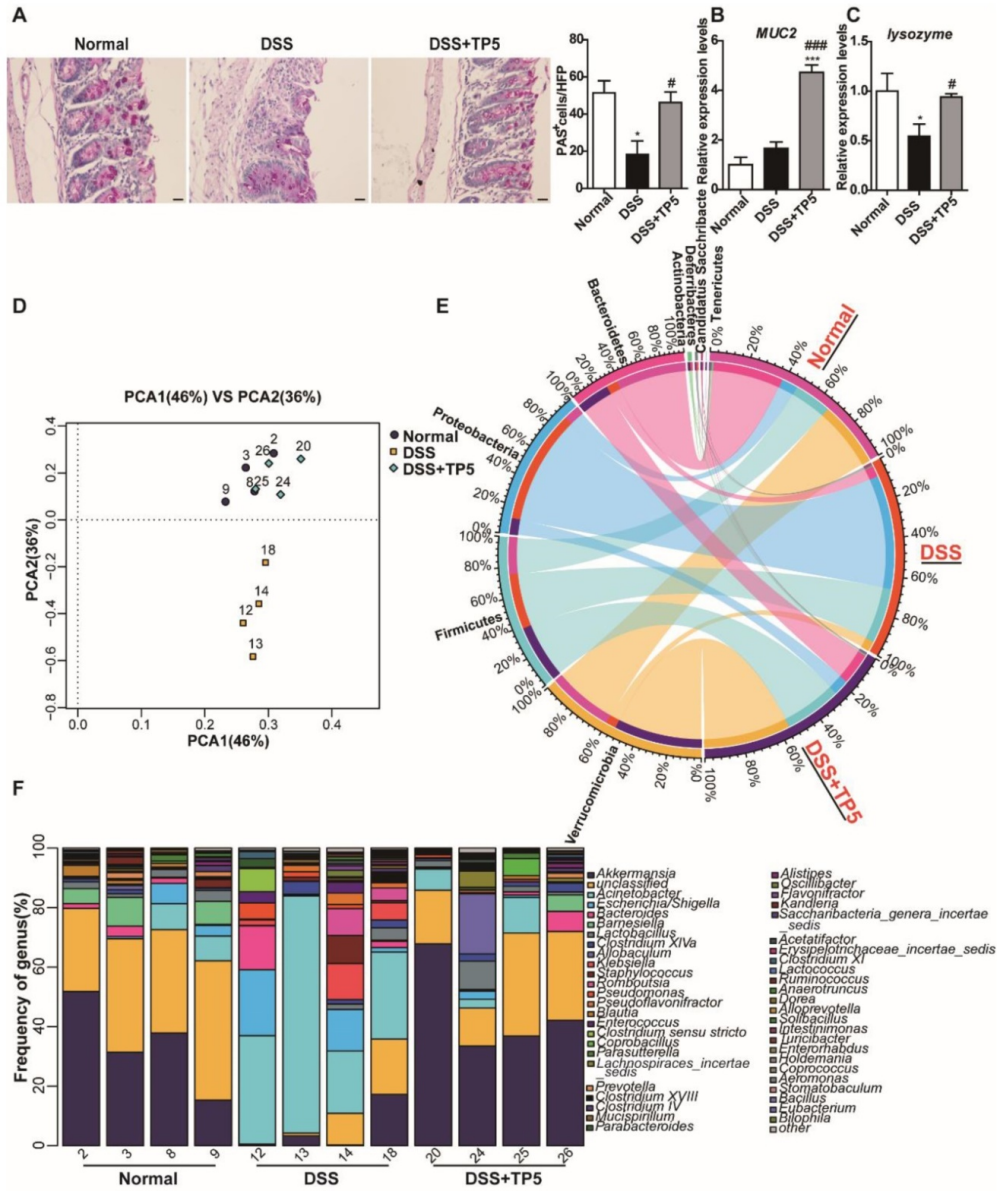
TP5 maintained the mucus barrier and normalized gut microbiota. (A) Representative PAS-stained goblet cell photographs and mean PAS^+^ goblet cells counted in 5 high power fields (HPF) in the colon. The scale bar represents 20 μm. (n=4-5). (B) RT-PCR results of *MUC-2* in the colon. (n=4-5). (C) RT-PCR results of *lysozyme* in the colon. (n=4-5). (D) PCA of microbiota in colon content. (n=4). (E) Circus data showed the composition of microbiota in different groups at the phyla level. (n=4). (F) Data from bar plots showed the composition of microbiota in mice treated with saline, DSS and DSS+TP5 at the genus level. (n=4). * *P* < 0.05, *** *P* < 0.001, compared with the normal group; # *P* < 0.05, ### *P* < 0.001, compared with the DSS group. DSS: dextran sulfate sodium; MUC2: mucin-2; PAS: periodic acid-schiff; PCA: principal component analysis; TP5: thymopentin.

Since IL-22 is primarily associated with the maintenance of mucus barrier function, we used Periodic Acid-Schiff (PAS) staining to assess the number of goblet cells which produce mucin. The results clearly showed restoration of the goblet cell number after TP5 treatment in colitic mice (Figure 5A). Consistent with this observation, TP5 also significantly increased the expression levels of *Mucin-2 (MUC2)* mRNA, which is the building block of colonic mucus (Figure 5B).

IL-22 is known to enhance the antibacterial defense of mucosal epithelial cells through different mechanisms 24, and UC is inextricably linked to the gut microbiome 25, 26. Also, the expression level of the antibacterial protein *lysozyme* was increased by TP5 (Figure 5C). We, therefore, assessed the composition of the gut microbiota in the colonic feces of each group. TP5 maintained the homeostasis of gut microbiota, completely protecting them from DSS, as shown by principal component analysis (PCA) data (Figure 5D).

The composition of microbiota at the phyla levels, as displayed in Figure 5E, indicates that the percentages of Verrucomicrobia, Bacteroidetes, and Actinobacteria were decreased sharply, whereas the percentages of Proteobacteria and Deferribacteres were increased in DSS-induced colitis. Consistent with the results of PCA, TP5 treatment abrogated the DSS-induced severe intestinal flora disturbance. To further confirm this result, we analyzed the composition of gut microbiota at the genus level. The results revealed that TP5 restored the greatly diminished composition of *Akkermansia*, *Acinetobacter*, *Barnesiella*, and *Lactobacillus*, and inhibited the markedly upregulated presence of *Escherichia/Shigella*, *Bacteroides*, *Klebsiella*, *Staphylococcus*, *Enterococcus,* and *Clostridium sensu stricto* in DSS-induced colitis (Figure 5F). Collectively, these results indicate that TP5 maintains the mucus barrier and normalizes gut microbiome, which may contribute to its protective effect on colitis.

### IL-22 mediated the protective effect of TP5

**Figure 6:**
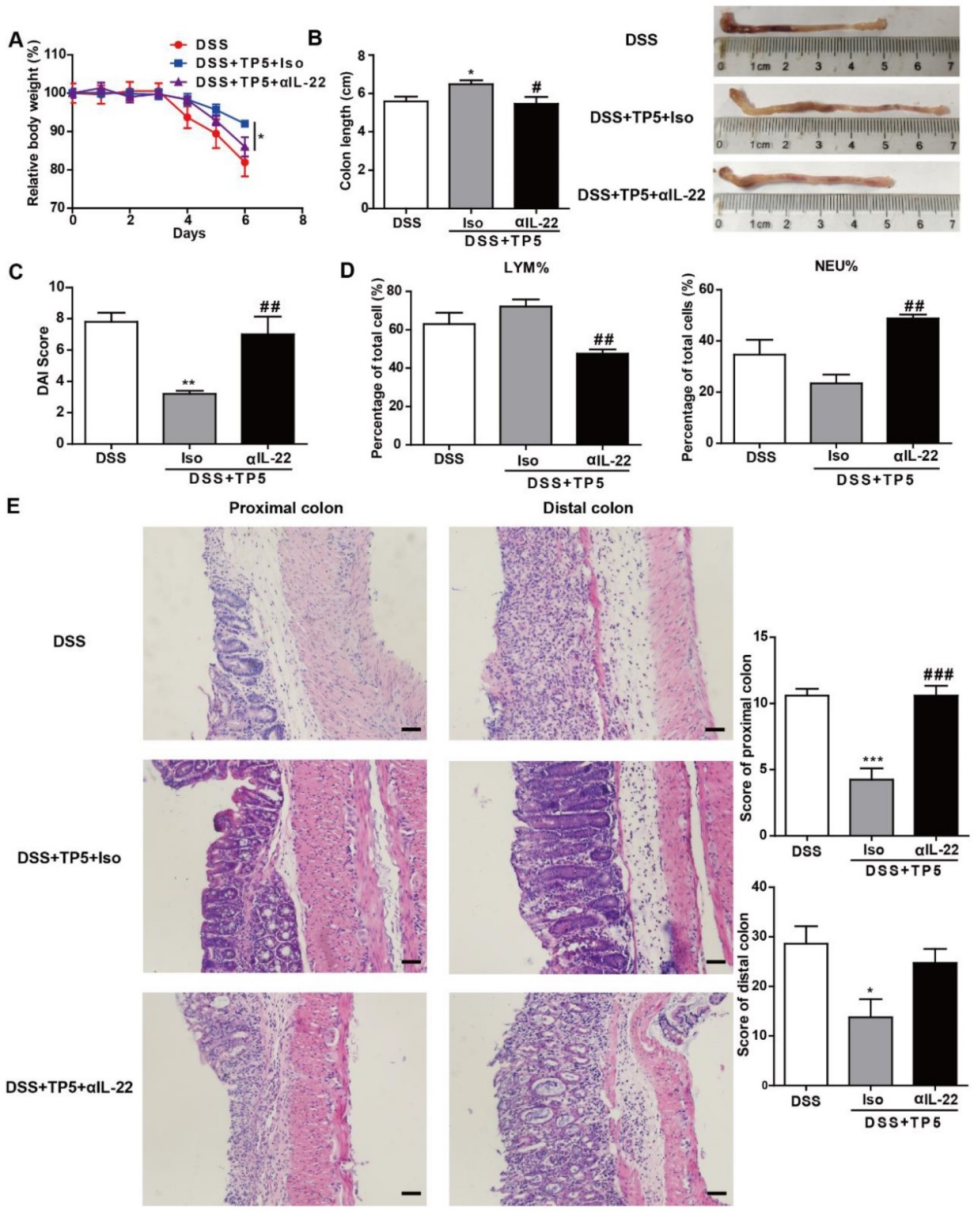
IL-22 mediated the protective effect of TP5 against DSS-induced colitis. (A-E) Mice were treated with DSS ± TP5 and given anti-IL-22 (αIL-22) or isotype control antibody (Iso) via i.p. once on day 0. (n=5). (A) Relative body weight curve of mice in each group. (B) Length of the colon and the representative colon photographs in each group. (C) DAI score. (D) Percentage of lymphocytes and neutrophils in whole blood. (E) Left: Representative H&E staining of proximal colon and distal colon. The scale bar represents 50 μm. Right: Histological score. * *P* < 0.05, ** *P* < 0.01, *** *P* < 0.001, compared with the DSS group; # *P* < 0.05, ## *P* < 0.01, ### *P* < 0.001, compared with the DSS+TP5+Iso group. αIL-22: anti-IL-22 antibody; Iso: isotype control antibody; DAI: disease activity index; DSS: dextran sulfate sodium; LYM: lymphocyte; TP5: thymopentin.

To investigate whether the protective effect of TP5 was mediated by IL-22, we treated the DSS-treated mice with saline, TP5 and anti-IL-22 antibody (αIL-22), and TP5 and isotype control antibody (Iso). Interestingly, the protective effect of TP5 was terminated by αIL-22, which also compromised the increased body weight and colon length in colitic mice treated with TP5 (Figure 6A-B). Also, αIL-22 abrogated the downregulation of DAI score in TP5-treated mice (Figure 6C). H&E staining revealed that αIL-22 blocked the protective effect of TP5 on colon inflammation and crypt damage of colitic mice (Figure 6E). Furthermore, in contrast to TP5+Iso, TP5+αIL-22 no longer upregulated LYM% or downregulated NEU% in the peripheral blood (Figure 6D). These results confirm that IL-22 mediates the protective effect of TP5 in DSS-induced colitis.

### TP5 upregulated IL-22 through RORγt

**Figure 7:**
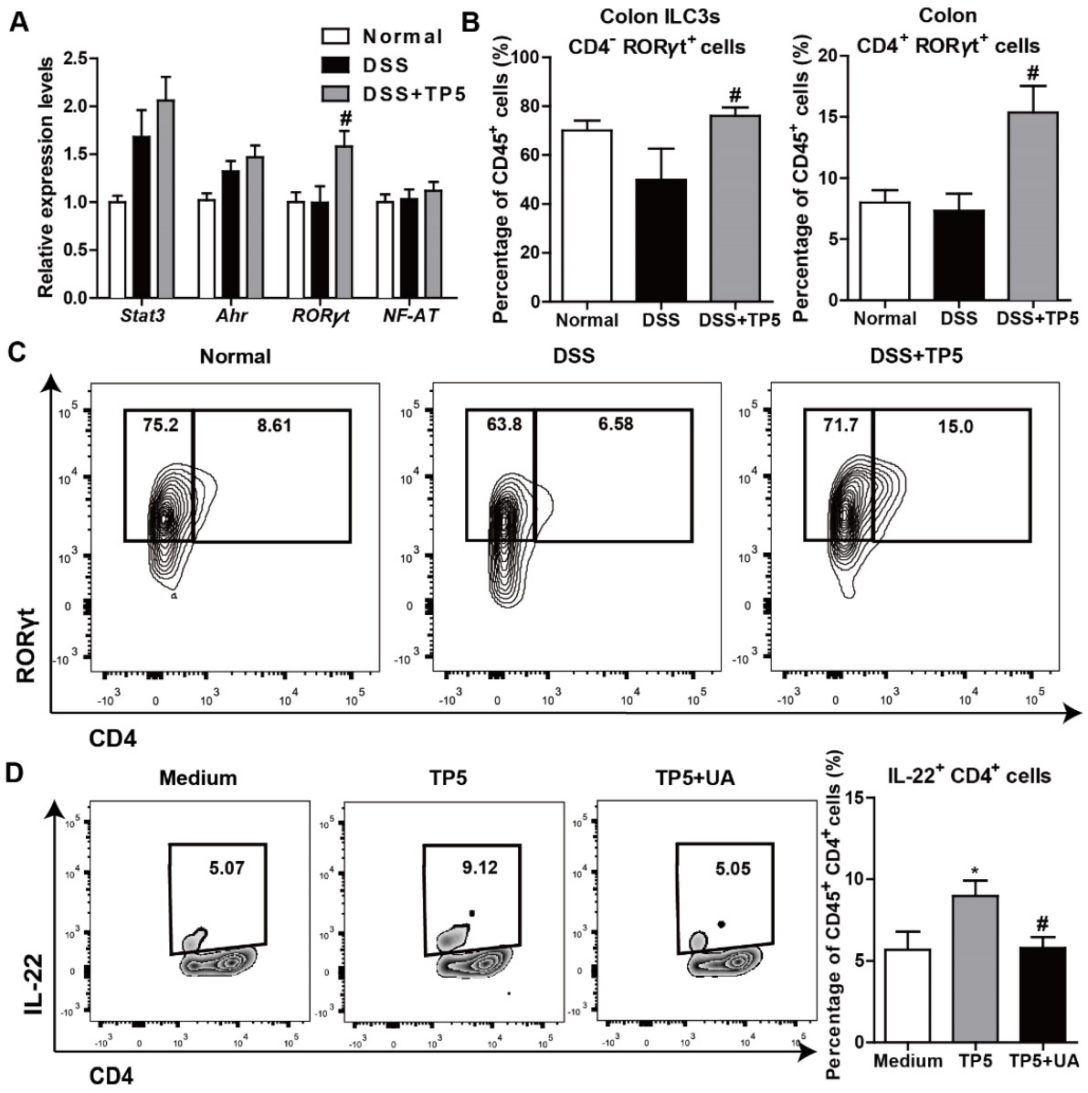
TP5 promoted IL-22 expression via RORγt. (A) RT-PCR results of *stat3, Ahr, RORγt,* and* NF-AT* in mouse colons. (n=4-5). (B-C) Flow cytometry analysis of ILC3s (CD45^+^, CD4^-^, RORγt^+^) and CD4^+^ RORγt^+^ cells (CD45^+^, CD4^+^, RORγt^+^) in colon LPMCs. (n=4-5). # *P* < 0.05, compared with the DSS group. (D) Flow cytometry analysis of IL-22 producing spleen T cells (CD45^+^, CD4^+^, IL-22^+^) treated with TP5 or ursolic acid (UA) *in vitro*. (n=6). At least two independent experiments were performed. * *P* < 0.05, compared with the medium group; # *P* < 0.05, compared with the TP5 group. DSS: dextran sulfate sodium; ILC3s: group 3 innate lymphoid cells; NF-AT: nuclear factor of activated T cells; TP5: thymopentin.

IL-22 can be produced by activated T cells and some subsets of innate lymphoid cells as previously described 24. Also, transcription factors, such as Stat3, aryl hydrocarbon receptor (Ahr), RORγt, and nuclear factor of activated T cells (NF-AT), were reported to regulate IL-22 expression 27, 28. To determine the mechanism of IL-22 expression promoted by TP5, we examined the regulatory factors of IL-22 in the colon samples from mice with or without treatment with TP5. We detected a significantly increased expression of RORγt in the DSS+TP5 group compared with DSS only group (Figure 7A). Similarly, we detected a higher number of CD45^+^ CD4^-^ RORγt^+^ (ILC3s) and CD45^+^ CD4^+^ RORγt^+^ cells in the colon lamina propria mononuclear cells (LPMCs) in DSS+TP5 group than in the DSS only group (Figure 7B-C). Ursolic acid (UA), a RORγt inhibitor 29, prevented the production of IL-22 in spleen T cells induced by TP5 (Figure 7D). These results suggest that RORγt plays an important role in the upregulation of IL-22 following TP5 treatment.

## Discussion

This study tried a new therapeutic strategy to combat UC, and found that TP5, a synthetic pentapeptide corresponding to the active site of thymopoietin, made a profound improvement on DSS-induced colitis. The immune-regulating reagent TP5 prevented thymic dysfunction in experimental colitis and restored the peripheral supply of lymphocytes. TP5 increased the expression level of IL-22, maintained the mucus barrier, and promoted normalization of the gut microbiota. Especially, TP5 promoted the production of IL-22 in both innate and adaptive lymphoid cells *in vitro*.

Though UC is known as a chronic inflammatory disease affecting the colon, it is accompanied with thymic involution 11, 19, 30. The thymus is known to be associated with colitis, as Tg_Ɛ_26 transgenic mice 31 and T-cell receptor α chain-deficient mice 32 develop colitis. In our study, we found a negative correlation between thymus coefficient and DAI score in colitic mice. Thymic involution causes suppression of the immune system, thereby increasing the incidence and severity of infections. TP5 promotes the differentiation of thymocytes, restores cyclophosphamide-induced suppression of the immune system, and has been clinically used for the treatment of immunodeficiency diseases 33, 34. Consistent with previous studies, we found that TP5 significantly abolished the thymic involution induced by DSS and restored the proportion of lymphocytes in blood and spleen accompanied by the remission of colitis.

Interestingly, although TP5 increased the proportion of peripheral lymphocytes, it decreased the percentage of inflammatory cells such as NEU in the peripheral blood. In the colon, the local inflammatory site, infiltration of macrophages and NEU were also decreased by TP5. Accordingly, pro-inflammatory cytokines, especially IL-6, were significantly diminished by TP5. The impaired inflammatory response was consistent with the protective effect of TP5 on the colon. These results negated concerns that immune enhancers like TP5 might increase inflammation. Although immune-suppressive drugs are widely used in the clinic for the treatment of UC, it is generally believed that the benefit of immunosuppressants is likely to be small while the toxicity is high 35, 36. On the other hand, immune-regulators such as TP5 might enhance the self-defensive ability of the body and thus mitigate the local inflammation of colitis.

To determine the underlying molecular mechanisms of the protective effect of TP5 on colitis, we focused on cytokines produced by lymphocytes, which play a central role in cell proliferation and differentiation, and defense against pathogens. Among several protective cytokines, we observed a marked increase of IL-22 expression by TP5 in colon and blood. IL-22 is a member of the IL-10 cytokine family and has emerged in recent years as a key effector molecule in host defense and in the pathogenesis of autoimmune diseases such as colitis 24, 37. The clinical relevance of IL-22/IL-22 receptor subunit 1 (IL-22R1) signaling system is being increasingly recognized in diseases such as psoriasis and UC 24. The upregulation of IL-22 induced by TP5 is consistent with the idea that it may serve as a promising therapeutic agent for inflammatory bowel disease (IBD) 38. Reports showed that IL-22 can be produced by activated T cells, including T helper 22 (T_H_22) cells, T_H_17 cells, T_H_1 cells, as well as subsets of innate lymphoid cells 39-42. To confirm the increased IL-22 induced by TP5 *in vivo*, we analyzed the production of IL-22 by TP5 *in vitro*. The results showed a significant increase in IL-22 expression in both innate and adaptive lymphoid cells. Most of these cells rely on specific transcription factors to promote the secretion of IL-22, for example, Stat3, Ahr, and RORγt 27. Our results also indicated a critical role of RORγt in TP5-induced increase of IL-22. Notably, the abolishment of IL-22 completely blocked the protective effect of TP5 on colitis. The data emphasize that increased IL-22 by TP5 promotes recovery of mice from DSS-induced injury. Furthermore, IL-22 is reported to drive endogenous thymic regeneration 43, and TP5 alleviated DSS-induced thymic atrophy, thus thymus could supply more T cells to peripheral tissues to maintain immune homeostasis after TP5 treatment, forming a positive feedback loop. TP5, of course, is the effect on the thymus more important, or the direct effect on the lamina propria lymphocytes, needs to be further investigated by thymectomized mice or nude mice.

We have demonstrated that TP5 protected colitis by producing IL-22 through RORγt/IL-22 signaling pathway, although how RORγt is induced by TP5 is not known. The receptor for TP5 has not yet been identified, and the downstream signaling pathway that directly interacts with TP5 is also not clear. It has been reported that T-cell receptor (TCR) 44 and Toll-like receptor (TLR2) 45 are the possible receptors of TP5. TP5 may bind directly to the membrane receptors on T cells and ILC3s to activate intracellular signaling pathways. We found that the expression level of Ahr in DSS+TP5 group was somewhat increased compared with the DSS group. Ahr is an important regulator in intestinal RORγt^+^ ILC and intraepithelial lymphocytes 46. Interestingly, both Ahr and RORγt transcription factors could directly bind to the promoter of IL-22. Ahr expression alone only marginally increased IL-22 expression, while RORγt alone induced IL-22 transcription and strong synergism was observed in cell lines transduced with RORγt and Ahr 46. Therefore, TP5 may directly bind to the membrane receptors on T cells and ILC3s and stimulate the Ahr/RORγt/IL-22 signaling pathway. Also, we found that the expression level of stat3 in DSS+TP5 group was higher than that in the DSS group. RORγt expression and T_H_17 differentiation are stat3-dependent 47, thus we speculated that TP5 may directly bind to the membrane receptors on T cells and ILC3s and activate the stat3/RORγt/IL-22 signaling pathway. However, how does TP5 actually regulate RORγt needs further investigation. We are trying to connect some reporters on TP5 and use some methods such as flow cytometry, Co-IP, mass spectrum etc. for further exploration.

Target cells of IL-22 are found in organs that mainly constitute the outer-body barriers such as the gastrointestinal system, but not in organs orchestrating immunity. IL-22 is known to act on mucosal tissues to regulate host defense responses. Mucus and antibacterial protein play essential roles in preventing and limiting bacteria, and both can be induced by IL-22 48, 49. Therefore, the increase of IL-22 by TP5 may explain its remarkable effect in restoring the number of goblet cells and MUC2 expression. The antibacterial protein lysozyme was also upregulated by TP5. It is of note that the markedly changed composition of the gut microbiome was abolished by TP5, which restored the composition of probiotics such as *Lactobacillus* and* Akkermansia,* known for its potential anti-inflammatory properties. Reduced levels of *Akkermansia* have been observed in patients with inflammatory bowel diseases (mainly UC) and metabolic disorders 50, 51. TP5 also decreased the composition of some proinflammatory and harmful bacteria, such as *Escherichia/Shigella*, *Bacteroides*, *Klebsiella*, *Staphylococcus*, and *Enterococcus*. These promising results of TP5 on the gut microbiome makeup further confirmed the therapeutic effect of TP5 on colitis. However, the modulation of the gut microbiome is highly complex and needs further characterization.

**Figure 8:**
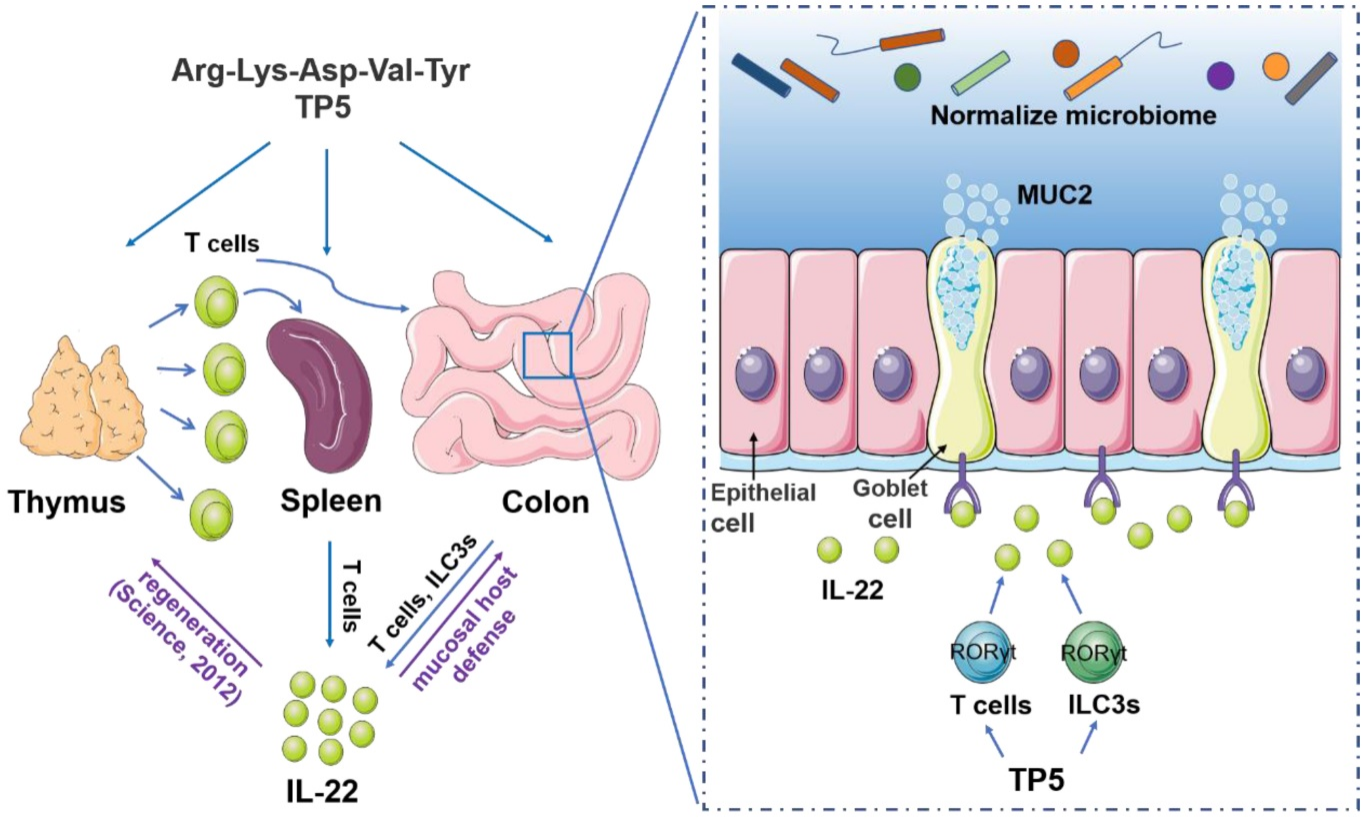
A proposed model illustrating the therapeutic effect of TP5 on DSS-induced colitis. TP5 promotes RORγt expression and triggers the production of IL-22 in both T cells and ILC3s. IL-22 then enhances the mucosal host defense in colon maintaining the mucus barrier and normalizing gut microbiome. Furthermore, IL-22 promotes the regeneration of damaged thymus thus generating more IL-22-producing T cells forming a positive feedback loop. ILC3s: group 3 innate lymphoid cells; MUC2: mucin-2; TP5: thymopentin.

Collectively, our results showed that TP5 increases the output of lymphocytes, elevates the expression of IL-22, normalizes the composition of the gut microbiome, and thus, alleviates DSS-induced colitis (Figure 8). Since thymus is a frequent target organ in a variety of diseases, this study not only posits an interesting concept that restoring an impaired thymus might be a useful therapeutic strategy for UC but also offers a therapeutic strategy for patients with the involuted thymus.

## Materials and Methods

**Mice.** Male C57BL/6N mice aged 6-7 weeks were purchased from Charles River (Beijing, China). Mice were house-caged with a 12 h/12 h light/dark cycle and habituated in the room 3 days before experiments. Animal experiments were conducted according to the National Institutes of Health Guide for the Care and Use of Laboratory Animals, with the approval of the Center for New Drug Safety Evaluation and Research, China Pharmaceutical University.

**Colitis induction with DSS.** Colitis was induced by 2.5% DSS (36-50 kD, MP Biomedicals, Canada) in the drinking water for 7 days. TP5 (HAINAN ZHONGHE PHARMACEUTICAL CO., LTD., Hainan, China. No: 20170906, 20180402, 20 mg/kg) was administrated by subcutaneous (s.c.) injection once every day during the time schedule and mice were weighed daily. On day 8, as previously reported 52, mice were sacrificed, and the length and weight of the whole colon were recorded after opening longitudinally and flushing with PBS. DAI scores were determined as previously described 53, 54. In brief, DAI was determined by scoring changes in weight, gross bleeding, and stool consistency. We used four grades of weight loss (0, no loss or weight gain; 1, 1% to 5% weight loss; 2, 5% to 15% weight loss; 3, more than 15% weight loss), four grades of stool consistency (0, normal; 1, slight loose; 2, loose; and 3, diarrhea), and four grades of gross bleeding (0, normal; 1, slight; 2, modest; and 3 severe). The combined scores constituted the final DAI. One-half of the mouse colons were used for flow cytometry analysis, and one half were divided into three sections (proximal, middle and distal). The proximal and distal colon sections were fixed in 4% phosphate-buffered formaldehyde for histological analyses. The middle colon was snap-frozen for subsequent molecular analyses. Mice treated with TP5 or saline for 7 or 14 days were sacrificed on day 8 and day 15, respectively. For αIL-22 treatment, the mice were administrated with one intraperitoneal (i.p.) dose of 400 µg anti-IL-22 (clone: IL22JOP, eBioscience, San Diego, USA) or 400 µg rat IgG2a κ isotype control (eBioscience, San Diego, USA), as previously described 55, and the mice were sacrificed on day 6 as some mice were dying.

**Histology and immunohistochemistry**. Proximal colon, distal colon, and the thymus tissues were fixed in 4% phosphate-buffered formaldehyde solution for 24 h and embedded in paraffin. Sections of 4 μm were stained with H&E. Colon inflammation and tissue damage were scored based on the degree of epithelial damage and inflammatory infiltrate in the mucosa, submucosa, and muscularis/serosa, as previously described 11, 56. Each of the four scores was multiplied by 1 if the change was focal, 2 if it was patchy, and 3 if it was diffuse. The H&E sections of thymus were evaluated and graded for the structure disorder and necrosis in thymus using a semiquantitative scale from 0 to 3. Cortical and medullary disorders and lymphocytic necrosis were scored as follows: mild=1, moderate=2, and severe=3, and the sum of the two scores were the total pathological score. For immunohistochemistry, after dewaxing and rehydration, the colon sections were soaked in sodium citrate buffer for heat-induced epitope retrieval, and incubated with 10% goat serum for 1 h to block the nonspecific binding sites. Subsequently, sections were incubated with anti-F4/80 antibody (1:200, CST, USA) or anti-Ki-67 antibody (1:100, CST, USA) overnight at 4℃, followed by incubation with horseradish peroxidase secondary antibodies for 20 min (MXB Biotechnologies, Fuzhou, China). The sections were stained using a Diaminobenzidine Substrate Kit (TIANGEN, Beijing, China) and counterstained with hematoxylin. Images were obtained with an Olympus BX41 microscope (Olympus, Japan).

**Cell isolation and flow cytometry.** Colonic LPMCs were isolated following a previously established method 57. In brief, luminal content, extraintestinal fat tissue, and blood vessels were removed, and colons were then cut into 0.5 cm pieces. The tissues were incubated in 20 mL of HBSS (Gibco, China) containing 5% FBS, 2 mM EDTA, and 1 mM DTT (Amresco, USA) at 37°C and predigested at 150 rpm for 20 min. The colon pieces were minced with gentle MACS^TM^ Octo Dissociator (Miltenyi Biotec, Germany) and digested in RPMI-1640 media (Biological Industries, Israel) containing 0.5 mg/mL Collagenase D (Roche, Mannheim, Germany), 0.5 mg/mL DNase I (Sigma, USA), and 3 mg/mL Dispase II (Sigma, USA). The digested cell suspension was then washed with PBS and filtered through a 70 μm cell strainer. Cells were stained with fluorescently conjugated antibodies: LIVE/DEAD™ fixable far-red dead cell stain kit (633 or 635 nm excitation, eBioscience, San Diego, USA), CD45 (BV510, Clone: 30-F11, Biolegend, USA), CD45 (FITC, Clone: 30-F11, Biolegend, USA), CD11b (PE, Clone: M1/70, Biolegend, USA), Ly6G (PE-CY7, Clone: 1A8, Biolegend, USA), CD3e (PE, Clone: 145-2C11, BD Biosciences, San Diego, USA), RORγt (APC, Clone: B2D, eBioscience, San Diego, USA), and F4/80 (APC, Clone: BM8, eBioscience, San Diego, USA).

Thymus homogenates or spleen homogenates after lysing red blood cells were also washed with PBS and filtered through the 70 μm cell strainer to get the cell suspension. Antibodies used for thymus staining included: LIVE/DEAD™ fixable violet dead cell stain kit (405 nm excitation, eBioscience, San Diego, USA), CD45 (FITC, Clone: 30-F11, Biolegend, USA), CD4 (APC-CY7, Clone: GK1.5, Biolegend, USA), and CD8 (PE-CY7, Clone: 53-6.7, Biolegend, USA). Antibodies used for spleen staining included: LIVE/DEAD™ fixable violet dead cell stain kit (405 nm excitation, eBioscience, San Diego, USA), CD45 (FITC, Clone: 30-F11, Biolegend, USA), CD19 (APC, Clone: 6D5, Biolegend, USA), CD3e (PE, Clone: 145-2C11, BD Biosciences, San Diego, USA), CD4 (APC-CY7, Clone: GK1.5, Biolegend, USA), CD8 (PE-CY7, Clone: 53-6.7, Biolegend, USA), CD11b (PE, Clone: M1/70, Biolegend, USA), Ly6G (PE-CY7, Clone: 1A8, Biolegend, USA), NK1.1 (APC, Clone: PK136, Biolegend, USA), and F4/80 (APC, Clone: BM8, Biolegend, USA). Cells were analyzed with the Invitrogen™ Attune™ NxT Flow Cytometer (Invitrogen, USA) or BD FACSVerse^TM^ Flow Cytometer (BD, USA) and were calculated using the FlowJo Software.

For the experiments with colonic LPMCs *in vitro*, we isolated fresh colons from normal C57BL/6N mice and used the methods mentioned above to obtain sterile LPMCs, also as previously reported 58, 59. The LPMC fractions were stimulated with TP5 (1 μg/mL) and Brefeldin A (4 μg/mL, MedChemExpress, Monmouth, USA) in RPMI-1640 supplemented with 10% fetal bovine serum (FBS, Biological Industries, Israel), 1% glutmax (Gibco, 100x, China), penicillin (100 U/mL, HyClone, USA), streptomycin (100 μg/mL, HyClone, USA) and 50 µM 2-mercaptoethanol (Sigma, USA) for 12 h. For the experiments with splenocytes *in vitro*, we isolated fresh spleens from normal C57BL/6N mice. The tissues were ground and dispersed into single cells with a sterile cell strainer (Corning, Durham, USA). The cell suspension was then lysed with red blood cell Lysis buffer (ebioscience, San Diego, USA) for 5 min. The remaining cells were counted and cultured after washing, as reported before 58, 59. The cells were cultured in the medium similar to LPMCs for 24 h except for Brefeldin A, and Brefeldin A was added for the last 4 h of culture. For some experiments, ursolic acid (2 μM, MedChemExpresss, Monmouth, USA), a RORγt inhibitor, was added to the spleen T cells. The cells were stained with the surface antibodies including LIVE/DEAD™ fixable far red dead cell stain kit (633 or 635 nm excitation, eBioscience, San Diego, USA), CD45 (BV510, Clone: 30-F11, Biolegend, USA), and CD4 (FITC, Clone: RM4-5, eBioscience, San Diego, USA), and were subsequently stained with RORγt (APC, Clone: B2D, eBioscience, San Diego, USA), IL-22 (PE, Clone: 1H8PWSR, eBioscience, San Diego, USA) after fixation and permeabilization (Cytofix/Cytoperm W/Golgi stop kit, BD Biosciences, San Diego, USA). Cells were detected by the BD FACSVerse^TM^ Flow Cytometer (BD, USA) and were analyzed by FlowJo Software.

**Real-Time PCR analysis.** Total RNA from colon tissues was extracted using RNeasy Plus Mini Kit (Qiagen, Germany), while cell cultures were extracted by using TRIzol (Invitrogen, San Diego, USA). Reverse transcriptase PCR (RT-PCR) was performed with a cDNA synthesis kit (Takara, China). Quantitative PCR was performed using SYBR Green QPCR Master Mix (Takara, China) with a Step one plus Real-Time PCR system (Applied Biosystems, USA). Relative amounts of mRNA were calculated by the ΔΔ*C*t method with β-actin as house-keeping genes. For the primer sequences, some of them were from PrimerBank (https://pga.mgh.harvard.edu/primerbank), others were designed with the Beacon Designer Software. All primers were custom-made by Genscript. The primer sequences were shown in Supplementary material.

**Inflammatory mediator measurement.** Colon tissues were weighed and homogenized using a tissue mixer (PRO Scientific Inc., USA) with 15 volumes of PBS. The tissue samples were then centrifuged at 3000 rpm for 20 min. Tissue supernatants were collected for the assays. IL-22 (Lianke Biotech CO., Ltd., Hangzhou, China), IL-1β, IFN-γ, TNF-α, and IL-6 (Dakewe Biotech Co., Ltd., Shenzhen, China) concentrations were measured by ELISA.

**Immunostaining of goblet cells.** For goblet cells immunostaining, tissue sections were dewaxed, hydrated, and stained with Periodic Acid-Schiff (PAS)/hematoxylin (SenBeiJia BioTech CO., Ltd., Nanjing, China). PAS^+^ goblet cells were counted in 5 different areas of the section, and at least 10 sections of each mouse. Measurement and observation were performed with an Olympus BX41 microscope.

**16S rRNA sequencing and analysis.** Colon content homogenates in PBS were immediately frozen (-80℃) and total community genomic DNA extraction was performed using an E.Z.N.A. Soil DNA Kit (Omega, USA), following the manufacturer's instructions. Next-generation sequencing library preparations and Illumina MiSeq sequencing were conducted at Sangon Biotech (Shanghai, China). The 16S rRNA V3-V4 amplicon was amplified using KAPA HiFi Hot Start Ready Mix (2×) (TaKaRa Bio Inc., Japan). Two universal bacterial 16S rRNA gene amplicon PCR primers (PAGE purified) were used: the amplicon forward PCR primer was 5'-CCTACGGGNGGCWGCAG-3' and the amplicon reverse PCR primer was 5'- GACTACHVGGGTATCTAATCC-3'. Sequencing was performed using the Illumina MiSeq system (Illumina MiSeq, USA), according to the manufacturer's instructions. The effective sequences of each sample were submitted to the RDP Classifier to identify archaeal and bacterial sequences.

**Cell culture and TP5 administration.** HCT116 cells were cultured in McCoy's 5A medium (Biological Industries, Israel) including 10% FBS, 100 U/mL penicillin and 100 μg/mL streptomycin. The medium of CT26 cells was RPMI-1640 containing 10% FBS, 100 U/mL penicillin and 100 μg/mL streptomycin. TP5 (0.01, 0.1, 1 μg/mL) was administrated for 1 and 3 days separately in the proliferation experiments, and 1 day for the RT-PCR experiments. Cell proliferation was measured using sulforhodamine B (Sigma, USA) assay, as previously described 60.

**Statistical Analysis**. Data are presented as mean ± SEM. Statistical significance was determined by two-tailed Student's t-test between two groups, and one-way ANOVA followed by Dunnett's posttests when groups were more than two. *P* < 0.05 was considered statistically significant.

## Abbreviations

αIL-22: anti-IL-22 antibody
Ahr: aryl hydrocarbon receptor
AIDS: acquired immune deficiency syndrome
BASO: basophil
Cldn2: claudin-2
DAI: disease activity index
DP: double positive
DSS: dextran sulfate sodium
EOS: eosinophil
FBS: fetal bovine serum
H&E: hematoxylin & eosin
HPF: high power field
IBD: inflammatory bowel disease
ILC3s: group 3 innate lymphoid cells
i.p.: intraperitoneal
Iso: isotype control antibody
LPMCs: lamina propria mononuclear cells
LUC: large unstained cell
LYM: lymphocyte
MONO: monocyte
MUC2: mucin-2
NEU: neutrophil
NF-AT: nuclear factor of activated T cells
Ocln: occludin
PAS: periodic acid-schiff
PCA: principal component analysis
PLT: platelet
RBC: red blood cell
RORγt: retinoic acid receptor-related orphan receptor γ
RT-PCR: reverse transcriptase PCR
s.c.: subcutaneous
SP: single positive
TCR: T-cell receptor
T_H_1: T helper1
T_H_17: T helper 17
T_H_22: T helper 22
Tjp1: tight junction protein 1
TLR2: Toll-like receptor
TP5: thymopentin
UA: ursolic acid
UC: ulcerative colitis
WBC: white blood cell
