# Supplementary material for: Thymopentin ameliorates dextran sulfate sodium-induced colitis by triggering the production of IL-22 in both innate and adaptive lymphocytes
Source: Theranostics. 2019 Oct 12;9(25):7490–505. doi: 10.7150/thno.35015 (PMC6831468; doi:10.7150/thno.35015)
Supplement: Supplementary file 1 — Supplementary figures and tables. [file thnov09p7490s1.pdf]

Supplementary Figures

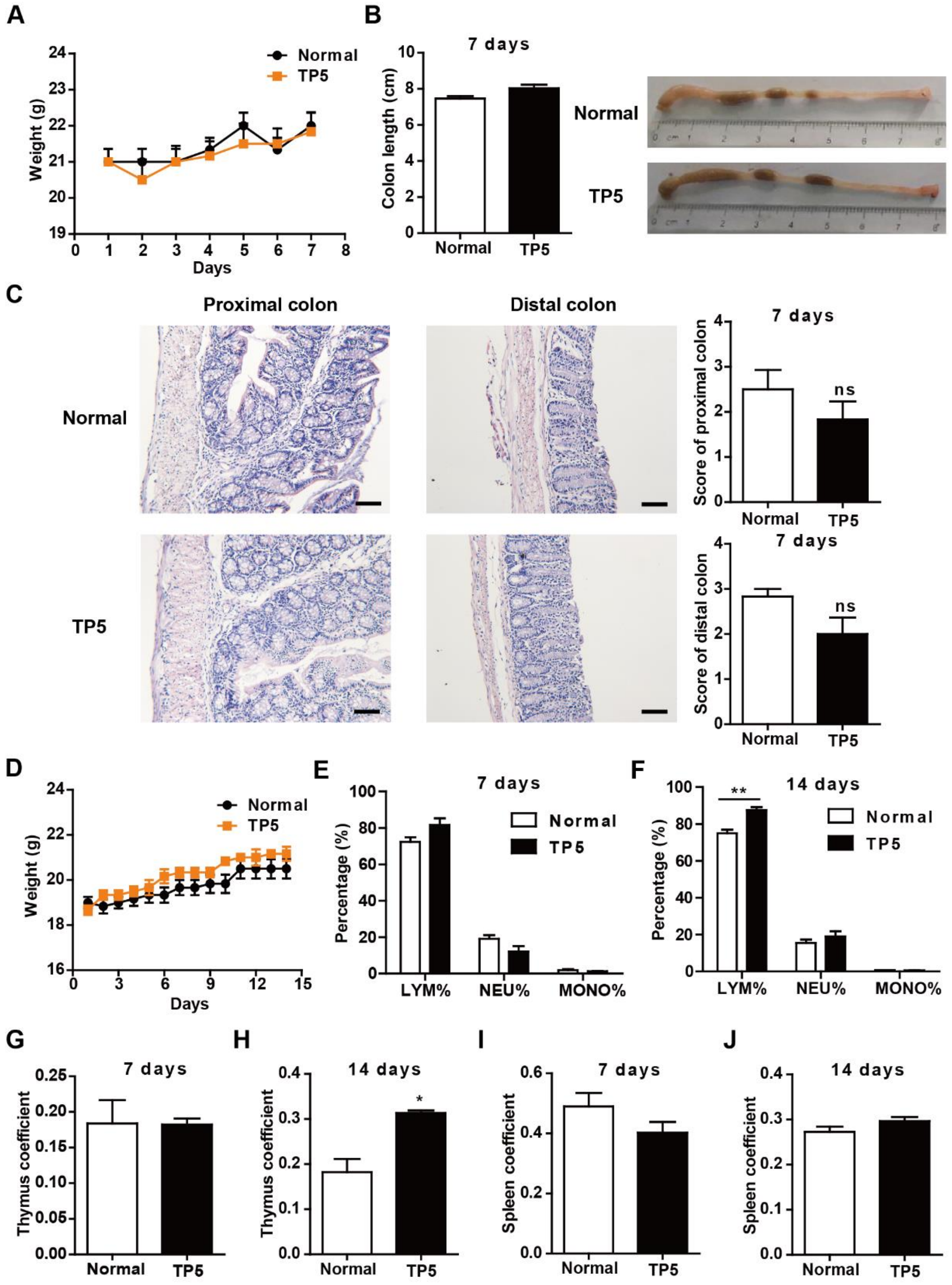

Figure S1. TP5 has no significant effect on the physiological function of normal mice. (A-C, E, G, I) The mice were treated with saline or 20 mg/kg TP5 for 7 days. (n=5). (A) Body weight curve of mice for 7 days. (n=5). (B) Colon length and representative colon photographs in the two groups. (C) Left: Representative H&E staining of proximal

colon and distal colon. Scale bar represents 50 μm. Right: Histological score. (D, F, H, J) Mice were treated with saline or 20 mg/kg TP5 for 14 days. (n=5). (D) Body weight curve of mice for 14 days. (E) Percentage of lymphocytes, neutrophils, and monocytes in peripheral blood in mice treated for 7 days. (F) The percentage of lymphocytes, neutrophils, and monocytes in peripheral blood in mice treated for 14 days. (G) Thymus coefficient of saline- or TP5-treated mice for 7 days. (H) Thymus coefficient of saline- or TP5-treated mice for 14 days. (I) Spleen coefficient of saline- or TP5-treated mice for 7 days. (J) Spleen coefficient of saline- or TP5-treated mice for 14 days. * $P < 0.05$, compared with the normal group. DSS: dextran sulfate sodium; LYM: lymphocyte; MONO: monocyte; NEU: neutrophil; TP5: thymopentin. At least two independent experiments were performed.

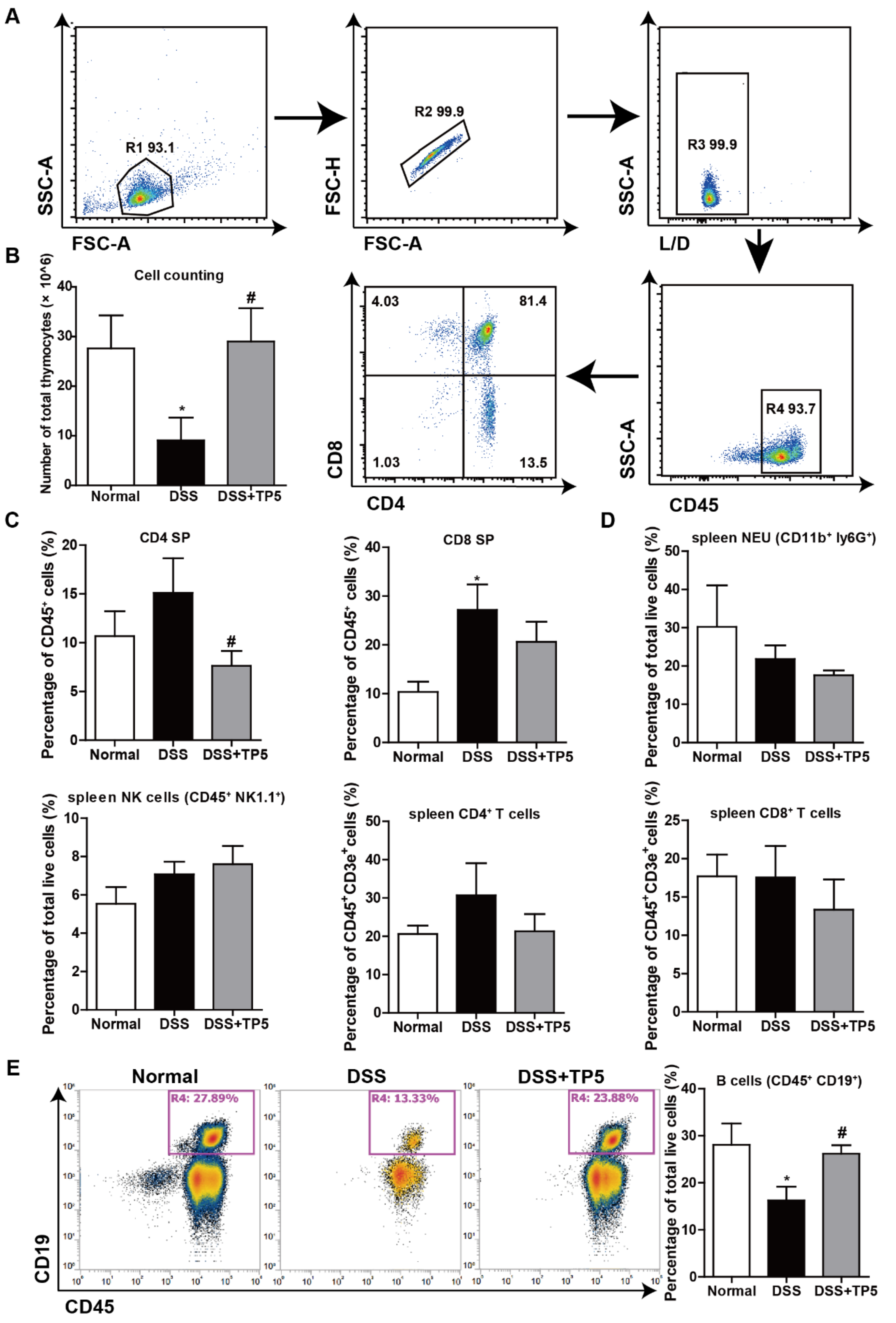

Figure S2. Immune cell analysis of the thymus and spleen. (A) Gating strategy for lymphocyte analysis by flow cytometry. (B) The numbers of total thymocytes detected

by cell counter. (n=4-5). (C) Flow cytometry analysis of CD4 single positive (CD4 SP) cells ($CD45^+$, $CD4^+$) and CD8 single positive (CD8 SP) cells ($CD45^+$, $CD8^+$) in the thymus. (n=4-5). (D) Flow cytometry analysis of NEU ($CD45^+$, $CD11b^+$, $Ly6G^+$), NK cells ($CD45^+$, $NK1.1^+$), CD4 T cells ($CD45^+$, $CD3e^+$, $CD4^+$), and CD8 T cells ($CD45^+$, $CD3e^+$, $CD8^+$) in the spleen. (n=4-5). (E) Flow cytometry analysis of B cells ($CD45^+$, $CD19^+$) in the spleen. (n=4-5). * $P < 0.05$, compared with the normal group; # $P < 0.05$, compared with the DSS group. DSS: dextran sulfate sodium; NEU: neutrophil; SP: single positive; TP5: thymopentin. At least two independent experiments were performed.

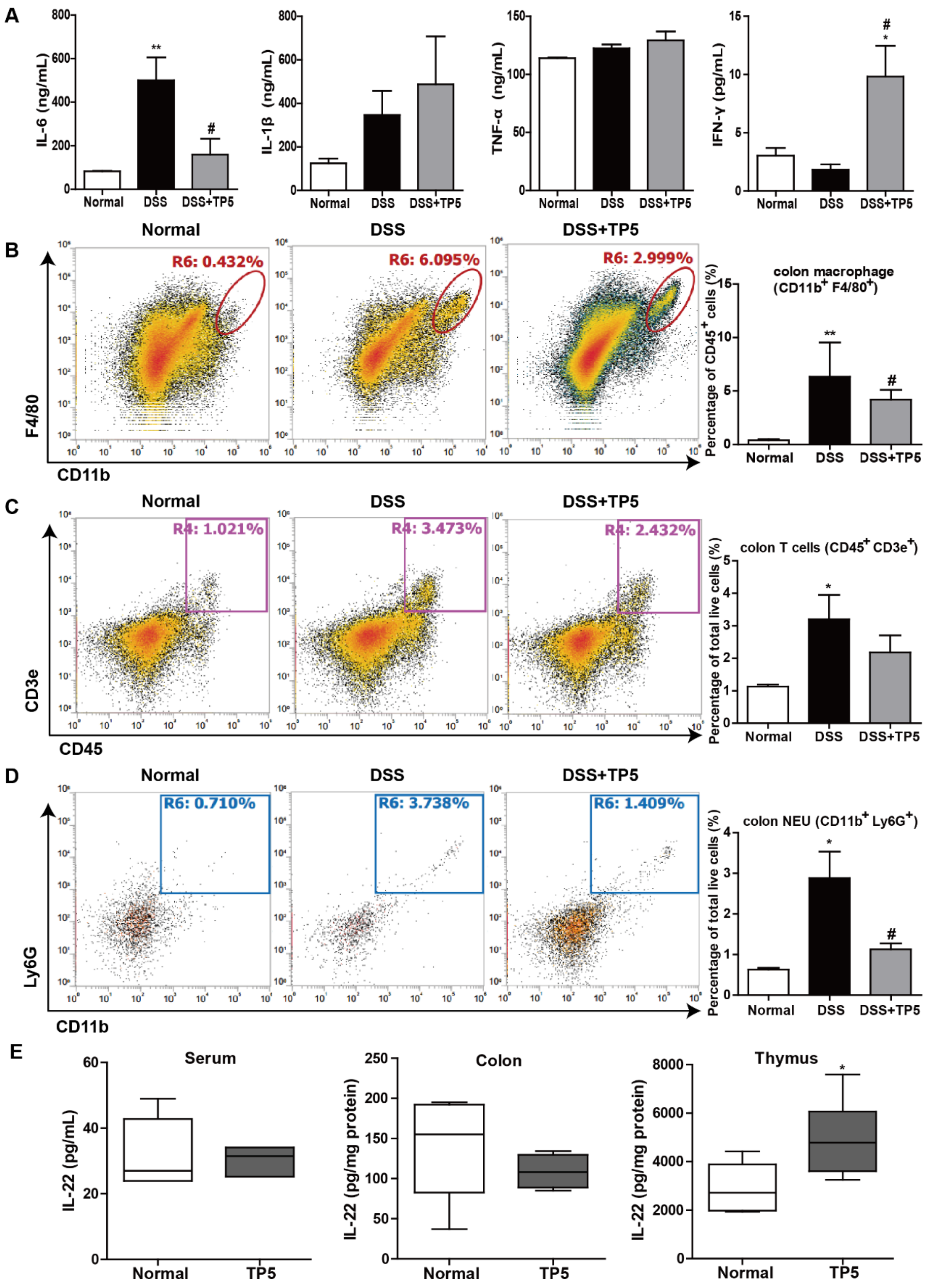

Figure S3. Detection of cytokines and inflammatory cells in the colon. (A) ELISA detection of IL-6, IL-1β, TNF-α, and IFN-γ in the colon. (n=4-5). (B-D) Flow cytometry analysis of macrophages ($CD45^+$, $CD11b^+$, $F4/80^+$), T cells ($CD45^+$, $CD3e^+$), and NEU

($CD45^+$, $CD11b^+$, $Ly6G^+$) in colon. (n=4-5). (E) Concentration of IL-22 in serum, colon, and thymus in mice treated for 14 days. (n=5). * $P < 0.05$, ** $P < 0.01$, compared with the normal group; # $P < 0.05$, compared with the DSS group. DSS: dextran sulfate sodium; NEU: neutrophil; TP5: thymopentin.

Table1. The primer sequences used for qPCR.

Gene		Sequence (5'to 3')
mTjp1	F	GCCGCTAAGAGCACAGCAA
	R	GCCCTCCTTTTAACACATCAGA
mOcln	F	TTGAAAGTCCACCTCCTTACAGA
	R	CCGGATAAAAAGAGTACGCTGG
mCldn2	F	CAACTGGTGGGCTACATCCTA
	R	CCCTTGGAAAAGCCAACCG
mIL-6	F	TAGTCCTTCCTACCCCAATTTCC
	R	TTGGTCCTTAGCCACTCCTTC
mIL-1β	F	CAATGGACAGAATATCAAC
	R	ACAGGACAGGTATAGATT
mIL-1α	F	CGAAGACTACAGTTCTGCCATT
	R	GACGTTTCAGAGGTTCTCAGAG
mTNF-α	F	TTCTGTCTACTGAACTTC
	R	CCATAGAACTGATGAGAG
mIL-18	F	GACTCTTGCGTCAACTTCAAGG
	R	CAGGCTGTCTTTTGTCAACGA
mIFN-γ	F	AGGCAGTATCACTCATTGT
	R	CAGCAGGTTATCATCATCATC
mCCL2	F	ATGAGATCAGAACCTACAACT
	R	TCCTACAGAAGTGCTTGAG
mIL-10	F	GGC CCA GAA ATC AAG GAG
	R	CCT TGT AGA CAC CTT GGT
mIL-23a	F	AATAATGTGCCCCGTATCCAGT
	R	GCTCCCCTTTGAAGATGTCAG
mIL-22	F	GAGGAGTCAGTGCTAAGG
	R	CATTCTTCTGGATGTTCTGG
mIL-12	F	ACATCTGCTGCTCCACAAG
	R	GGTGCTTCACACTTCAGGAA
mTGF-β	F	GGATACCAACTATTGCTTCAG
	R	TGTCCAGGCTCCAAATATAG
mMUC2	F	ACCATTACCACCACTACAG
	R	CAGGAGCACTACAGACAT

Gene		Sequence (5'to 3')
mlysozyme	F	GAGACCGAAGCACCGACTATG
	R	CGGTTTTGACATTGTGTTCGC
mRORγt	F	GACCCACACCTCACAAATTGA
	R	AGTAGGCCACATTACACTGCT
mNF-AT	F	TCATCCAACAACAGACTGCCC
	R	GGGAGGGAGGTCCTGAAAACT
mstat3	F	AGCTGGACACACGCTACCT
	R	AGGAATCGGCTATATTGCTGGT
mAhr	F	AGCCGGTGCAGAAAACAGTAA
	R	AGGCGGTCTAACTCTGTGTTC
mβ-actin	F	ACCACACCTTCTACAATGAG
	R	ACGACCAGAGGCATACAG
hTjp1	F	ACCAGTAAGTCGTCCTGATCC
	R	TCGGCCAAATCTTCTCACTCC
hβ-actin	F	GCGTGACATTAAGGAGAAG
	R	GAAGGAAGGCTGGAAGAG
